# Exploring the coupling dynamics between accessibility of tourist attractions and tourism demand in Anhui Province

**DOI:** 10.1371/journal.pone.0331577

**Published:** 2025-09-03

**Authors:** Jilei Tao, Hong Wei, Qinan Wang, Xiulong Jin

**Affiliations:** 1 School of Geographic Information and Tourism, Chuzhou University, Chuzhou, China; 2 Anhui Province Key Laboratory of Physical Geographic Environment, Chuzhou University, Chuzhou, China; 3 Anhui Engineering Laboratory of Geo-Information Smart Sensing and Services, Chuzhou, China; Macau University of Science and Technology, MACAO

## Abstract

Tourist attractions, as core components of tourism product supply, play a crucial role in regional tourism development. Investigating the spatial relationship between the supply and demand of tourist attractions contributes to optimizing the spatial structure of regional tourism and promoting high-quality, balanced, and coordinated growth of the tourism industry. Taking high-grade tourist attractions in Anhui Province as the research object, this paper first analyzes the characteristics of accessibility using the time cost distance method and the standard deviational ellipse. Subsequently, it constructs a set of tourism demand indicators and employs the coupling coordination model and Markov chain method to evaluate the spatial coupling coordination between accessibility of tourist attractions and tourism demand. The results show a significant improvement in accessibility of tourist attractions from 2015 to 2024, with average travel time to tourist attractions at the county level decreasing from 65 to 41 minutes, and the proportion of areas reachable within 60 minutes rising from 62.5% to 92.3%. However, spatial disparities persist, particularly exhibiting persistent low accessibility in mountainous and peripheral areas. The coupling coordination levels between the accessibility of tourist attractions and tourism demand remained generally stable, with most counties classified into the moderate and high coordination categories. Spatial Markov analysis indicates that neighboring regions exert strong spatial spillover effects, influencing both the direction and magnitude of coordination level transitions. Notably, leap-level transitions were rare, while adjacent-level shifts dominated the dynamics. These findings underscore the importance of spatial context and infrastructure in shaping the interaction between tourism supply and demand. This study provides empirical evidence to support spatially differentiated planning and transportation investment strategies aimed at promoting balanced tourism development.

## 1. Introduction

As global tourism develops rapidly, the tourism industry has become a key driver of economic growth, cultural exchange, and heritage preservation. Tourist attractions, as important spatial carriers of tourism activities [1], are both a core component of tourism product supply and a key factor in generating travel motivation [2,3]. With China’s social and economic progress, public demand for tourism has surged, making tourist attractions crucial for boosting local tourism economies and enriching visitors’ cultural experiences [4,5]. According to the Statistical Bulletin on Cultural and Tourism Development (2024) issued by the Ministry of Culture and Tourism, by the end of 2024, China had a total of 16,541 A-level tourist attractions, providing direct employment for approximately 1.653 million people. Over the course of the year, these attractions received a total of 6.76 billion tourist visits and generated tourism revenue of 481.42 billion yuan.

Research on tourist attractions began in the 1960s [6]. Some studies suggest that in regions where the supply of tourist attraction services exceeds demand, resources are not fully utilized; conversely, in areas where supply falls short of demand, tourist satisfaction declines and travel intention weakens [7], ultimately impeding the overall development of regional tourism. Numerous scholars have explored the supply–demand relationship of tourist attractions. In demand-oriented studies, researchers have extensively analyzed the characteristics, behavioral preferences, and influencing factors of tourists [8]. On the supply side, studies have explored the subjects, content, models, and development trends of tourism service provision [9]. Research on supply–demand relationships has revealed the manifestations, impacts, and regulatory strategies of both equilibrium and imbalance [10]. Although existing research has laid a solid theoretical and methodological foundation for follow-up studies, several limitations remain. Traditional studies on supply and demand in tourist attractions often rely on questionnaires and interviews, and the lack of Geographic Information System (GIS) applications limits the ability to detect spatial patterns in supply-demand mismatches, reducing the effectiveness of findings in optimizing tourism transportation and attraction layout [11,12].

As an important indicator for measuring the supply level of spatial geographic elements, accessibility has been widely applied in studies on the supply–demand relationship of resources such as medical institutions [13,14], educational facilities [15,16], and urban green spaces [17,18]. Some scholars have also applied this concept to the study of tourist attractions. Accessibility of tourist attractions refers to the ease or difficulty with which tourists can reach attractions [19]. It is a key factor influencing the scale of tourist source markets and the regional tourism economic linkages, and thus occupies an important position in tourism research [20]. Existing studies on the accessibility of tourist attractions primarily focus on measuring accessibility levels, analyzing influencing factors, and formulating strategies to enhance accessibility [21,22]. In terms of research scale, studies span national, provincial, urban, and individual scenic area levels [23]. Commonly used research methods include buffer analysis, minimum nearest neighbor distance, the two-step floating catchment area (2SFCA) method, and time cost distance method [24].

Nonetheless, several critical issues remain unresolved in current accessibility of tourist attractions research. First, most existing studies examine the accessibility of tourist attractions and tourism demand independently, with limited attention to the intrinsic connection and dynamic coupling relationship between the two. Second, the accuracy of time cost calculations in accessibility assessment requires improvement. While existing research primarily considers traffic-related factors, it rarely accounts for natural environmental constraints. Moreover, comparative analyses of the temporal variation in accessibility characteristics remain limited. Given that traffic conditions, the spatial distribution of attractions, and socio-economic factors evolve continuously, exploring the spatiotemporal evolution of accessibility is crucial for identifying development patterns and long-term trends.

**Fig 1:**
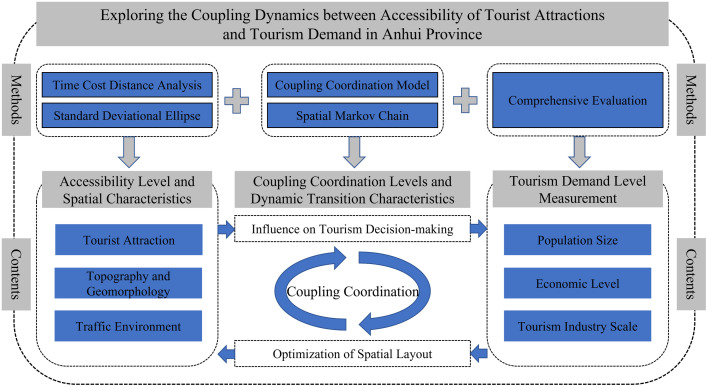
Roadmap of the technical approach.

Building on this, and by comprehensively considering factors such as traffic conditions, the natural environment, and socio-economic elements, this study employs the time cost distance method, the coupling coordination model, and the Markov chain model to analyze the spatiotemporal coupling coordination characteristics between the accessibility of high-grade tourist attractions and tourism demand in Anhui Province. This study aims to provide a reference for promoting the sustainable and coordinated development of the tourism industry in Anhui Province. The overall research framework is illustrated in Fig 1.

## 2. Materials and methods

### 2.1. Study area

**Fig 2:**
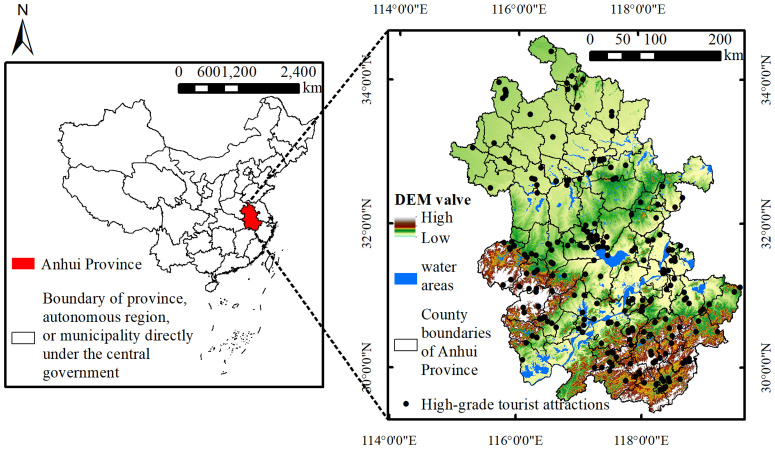
Scope of the study area.

Anhui Province is located in the central-eastern region of China and serves as a key part of the Yangtze River Delta Economic Zone. Its specific location is illustrated in Fig 2. The province borders the Yangtze River and connects to coastal areas, featuring diverse terrain and a pleasant climate. By the end of 2024, Anhui comprised 16 prefecture-level cities and 104 county-level administrative units, with a permanent population of 61.23 million [25]. The total length of expressways reached 6,153 kilometers, while the operational railway network extended to 5,737 kilometers [26]. The province hosted 668 A-level tourist attractions, including 13 5A-level, 213 4A-level, and 442 3A-level or below attractions. In 2023, inbound tourist visits reached 221,170, while domestic tourist visits totaled 848.44 million. Foreign exchange earnings from tourism amounted to USD 227.19 million, and domestic tourism revenue reached 850.96 billion yuan [27]. Tourism revenue accounted for approximately 18.4% of the provincial GDP, underscoring the industry’s significant role in Anhui’s economic development. However, due to variations in natural and cultural environments across regions of the province, the spatial distribution of tourist attractions remains highly uneven, particularly in the case of high-grade attractions (i.e., 4A- and 5A-level), which require high resource value and comprehensive service quality [28]. Therefore, it is urgent to examine the spatial coupling relationship between tourist attraction accessibility and tourism demand, in order to inform spatial planning for the province’s overall tourism development.

### 2.2. Data sources and preparation

The vector data used in this study, including water systems, and road network data (2015) were obtained from the National Geographical Information Resource Directory Service System (https://www.webmap.cn). The 2024 road network data were sourced from the OpenStreetMap database (https://www.openstreetmap.org). Elevation and slope data were derived from 90-meter resolution datasets provided by the Geospatial Data Cloud (https://www.gscloud.cn). The administrative boundary data of the study area were generated through vectorization and spatial correction. Data on permanent population and per capita GDP were collected from statistical yearbooks and statistical bulletins on national economic and social development at the provincial, municipal, district, and county levels. Information on the number of travel agencies and hotels was obtained from the Qichacha platform (https://www.qcc.com). Data regarding the classification, locations, and evaluation dates of high-grade tourist attractions were sourced from the Directory of A-level Tourist Attractions in Anhui Province, published on the official website of the Anhui Provincial Department of Culture and Tourism in 2024. This study focuses specifically on high-grade tourist attractions, namely 4A- and 5A-level sites, for the following reasons. First, China’s 4A- and 5A-level tourist attractions are officially designated by provincial or national tourism administrative authorities and are subject to strict evaluation criteria regarding resource landscape value and service quality [29,30]. These tourist attractions are central to the development of regional tourism and thus hold significant research value [31]. Second, data and documentation related to high-grade tourist attractions are relatively complete, facilitating both reliable data acquisition and accurate spatial positioning.

### 2.3. Research methods

#### 2.3.1. Spatial accessibility analysis.

This study calculates the spatial accessibility of high-grade tourist attractions in Anhui Province using the time cost distance method. Compared with other accessibility analysis approaches, this method is well-suited for assessing the accessibility of geographical elements at large spatial scales [32]. It comprehensively incorporates terrain, landforms, and the traffic environment, transforming spatial accessibility into time-based accessibility [33]. This approach enables dynamic simulation of human travel behavior, thereby avoiding the limitations of static spatial analysis. The cost distance analysis tool in ArcGIS 10.2 was used to calculate accessibility, taking full account of travel costs associated with various transportation networks and topographical conditions in the region. The main steps of the method are as follows:


$$T=\frac{c\times 60}{v\times 1000}$$ (1)

In the formula, *T* represents the travel time cost (in minutes), *c* is the length of a grid cell edge (in meters), which is set to 1000 meters, and *v* denotes travel speed (in kilometers per hour). Reference values for the time costs of different road types are primarily based on the Highway Engineering Technical Standards of the People’s Republic of China. The corresponding speed values assigned to different road classes are listed in Table 1.

**Table 1:** Speed and time cost for different road types.

Road types	High-speed railway	Ordinary railway	Expressway	Class I or arterial road	Class II or collector road	Class III or local road	Class IV or rural road
Traffic speeds (km/h)	250	100	120	80	60	40	30
Time cost (min/km)	0.24	0.6	0.5	0.75	1	1.5	2

Simultaneously, under varying topographical conditions, human activities must overcome different levels of spatial resistance; therefore, travel time costs should vary accordingly [21]. In this study, slope and relief are categorized, with corresponding travel time costs assigned to each [22]. Areas traversed by rivers and lakes are considered impassable by default and are assigned a high time cost of 100 minutes [24], as shown in Table 2.

**Table 2:** Time cost of natural environment.

	Rank (°)	Time cost (min/km)		Rank(m)	Time cost (min/km)		Time cost (min/km)
Slope	<5	12	relief	<15	12	Lakes and rivers	100
	5 ~ 15	18		15 ~ 30	15		
	15 ~ 25	30		30 ~ 60	18		
	>25	50		>60	30		

The time cost distance analysis uses the spatial distribution layer of high-grade tourist attractions in Anhui Province as the source feature and the composite time cost raster layer as the cost surface. It calculates the time distance from each raster cell to the nearest high-grade tourist attraction. The specific formula is as follows:


$$A_{i}(\text{min})=\left\{\begin{array}{c} {}*20c\frac{1}{2}\sum_{i=1}^{n}\left(T_{i}+T_{i+1}\right)\\ \frac{\sqrt{2}}{2}\sum_{i=1}^{n}\left(T_{i}+T_{i+1}\right) \end{array}\right\}$$ (2)

In the formula, *A_i_* represents the time cost of raster *i*, indicating the time (in minutes) required to travel from raster *i* to the nearest tourist attraction. *T_i_* is the cost value (in minutes) of raster *i*, and *T_i_* ₊ ₁ is the cost value (in minutes) of raster *i* + 1. n denotes the total number of raster cells. A smaller value of *A_i_* implies a shorter travel time to the attraction, thereby indicating better accessibility.

County-level administrative units are a fundamental component of China’s administrative system and play a key role in the country’s political and economic activities. Therefore, using counties as the basic spatial statistical units for zonal statistical calculation and classification of the accessibility of high-grade tourist attractions in Anhui Province is conducive to providing a valuable data reference for regional tourism development planning and tourism resource integration. The calculation formula is as follows:


Rj=∑i=1n\nolimitsj\raise0.7ex\({{A_i\)/Ainj\nulldelimiterspace\lower0.7ex\({{n_j}\)}}} (3)

In the formula, *R_j_* represents the accessibility of county-level administrative unit *j*; *n_j_* denotes the number of raster cells within unit *j*; and *A_i_* is the accessibility value of the tourist attraction in raster *i*. A smaller value of *R_j_* indicates better overall accessibility of tourist attractions in that administrative unit.

#### 2.3.2. Standard deviational ellipse.

The standard deviational ellipse (SDE) is employed in this study to examine the spatial morphological characteristics of the accessibility of high-grade tourist attractions in Anhui Province, including agglomeration, directionality, and centrality [34]. In the ellipses generated by the analysis, the major and minor axes, along with the azimuth angle, represent the extent and orientation of the accessibility time distribution. The area of the ellipse reflects the degree of spatial agglomeration, while its center indicates the spatial centroid of the accessibility time distribution for tourist attractions.

#### 2.3.3. Coupling coordination model.

The coupling coordination model is derived from the concept of coupling in physics and serves as an important method for assessing the degree of interconnection between systems or elements. It has been widely applied in fields such as sociology, economics, and other disciplines [35]. In this study, the model is employed to evaluate the level of coupling and coordinated development between the accessibility of tourist attractions and tourism demand across counties in Anhui Province. Population size, per capita GDP, and the number of travel agencies and hotels are selected as key indicators to reflect tourism demand in each region. The rationale for selecting these indicators is elaborated in the corresponding section. The tourism demand level is assessed through a comprehensive evaluation based on standardized indicators and equal-weighted averaging, and the specific calculation process is as follows:

The accessibility of tourist attractions and the tourism demand indicators were normalized separately. Specifically, higher values of indicators such as population size, per capita GDP, number of travel agencies, and number of hotels indicate higher tourism demand in a region. These indicators were standardized using a positive normalization method. In contrast, a shorter travel time to a tourist attraction indicates higher accessibility, the time of accessibility was standardized using a reverse normalization method. The specific formulas are as follows:


$$X_{ij}=\frac{x_{ij}-x_{\min}}{x_{\max}-x_{\min}}$$ (4)

$$Y_{i}=\frac{y_{\max}-y_{i}}{y_{\max}-y_{\min}}$$ (5)

In the formula, *X*_*ij*_ represents the standardized value of indicator *j* in region *i*, and *x*_*ij*_ represents the original value of indicator *j* in region *i*. *Y*_*i*_ represents the standardized value of tourist attraction accessibility in region *i*, and *y*_*i*_ represents its original value. Calculate the system coupling degree as follows:


$$C_{i}=2\sqrt{\frac{U_{i}E_{i}}{{\left(U_{i}+E_{i}\right)}^{2}}}$$ (6)

In the formula, *C*_*i*_ is the system coupling degree; *U*_*i*_ is the accessibility level of tourist attractions in each region; and *E*_*i*_ is the tourism demand level of tourist attractions in each region.

Calculate the coupling coordination degree using the following formula:


$$D_{i}=\sqrt{C_{i}⬝T_{i}}$$ (7)

$$T_{i}=\alpha U_{i}+\beta E_{i}$$ (8)

In the formula, *C*_*i*_ is the degree of coupling. *D*_*i*_ is the degree of coupling coordination. *T*_*i*_ is the comprehensive coordination index; *α* and *β* are the supply and demand weight coefficients, reflecting the respective importance of supply and demand (*α* + *β* = 1), and the values of *α* and *β* in the article are 0.5.

#### 2.3.4. Markov chain.

Markov chain analysis is an important method for revealing the spatiotemporal dynamics of regional phenomena. Compared with spatial statistical methods such as the Terre index and the coefficient of variation, it can directly reflect the dynamic evolution process of regional systems and internal state transitions [36]. Markov chain analysis can be categorized into traditional and spatial variants. To investigate whether the coupling relationship between the accessibility of high-grade tourist attractions and tourism demand in Anhui Province is influenced by spatial spillover effects and agglomeration patterns, this study first employs a traditional Markov process. The continuous values of coupling coordination levels between accessibility and tourism demand are discretized into *k* categories. A 1 × *k* matrix *F*_*t*_ = [*F*_1_, *t*, *F*_2_, *t*, ⋯, *F*_*k*_, *t*] is constructed to represent the probability distribution of the system’s state at time *t*. The transition of states across different time periods is represented by a *k* × *k* matrix M. On this basis, to account for the influence of spatial geographic factors, the traditional *k* × *k* Markov matrix is further decomposed into *k* conditional *k* × *k* transition probability matrices by introducing the concept of spatial lag as a conditional variable.

## 3. Results

### 3.1. Evolution of the spatial patterns of tourist attractions accessibility

#### 3.1.1. Characterization of the overall spatial distribution of accessibility.

**Fig 3:**
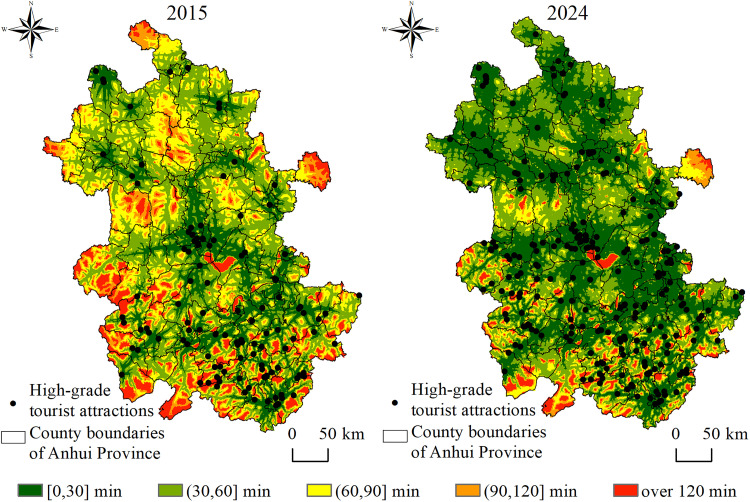
Spatial distribution of accessibility to tourist attractions at the grid scale.

**Fig 4:**
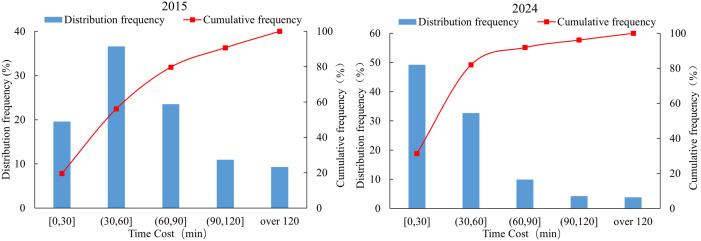
Frequency of accessibility time distribution of tourist attractions at the grid scale.

Based on the time cost distance method, spatial accessibility distribution maps and accessibility time frequency distribution maps at the grid scale were generated for Anhui Province in 2015 and 2024 (Figs 3 and 4). Compared with 2015, the spatial accessibility of high-grade tourist attractions in 2024 exhibits the following distribution characteristics and trends at the grid level.

Significant overall improvement in tourist attractions accessibility. In 2015, the average time to high-grade tourist attractions in Anhui Province was 65 minutes. Grids with travel times exceeding 60 minutes were widely distributed, accounting for approximately 43% of the total area. By 2024, the average time had decreased to 41 minutes. Grids with short travel time expanded substantially, while those with long travel time contracted markedly. The proportion of grids with the time of accessibility within 60 minutes increased to 81%.

Reduced regional disparity in tourist attractions accessibility. In 2015, parts of northern and southern Anhui were constrained by factors such as tourism resources, topography, and transportation infrastructure, resulting in a pronounced clustering of grids with long travel time and a clear “island effect” in tourist attractions accessibility. By 2024, with intensified tourism development, increased expressway density, and the advancement of rapid transport corridors, accessibility conditions in traditionally low-accessibility areas such as provincial border regions and mountainous-hilly transition zones had substantially improved.

Tourist attractions accessibility in certain local areas remains poor. Despite the substantial optimization of accessibility across the province, scattered point-like and patchy grids with long travel time still persist along some provincial borders and in parts of the southern mountainous regions. These patterns reflect the limitations in the extension of terminal transportation networks. Constrained by geographic location and high construction costs, these areas have experienced relatively lagging improvements in tourist attractions accessibility, forming “marginal zones”.

In summary, the spatial pattern of accessibility to high-grade tourist attractions in Anhui Province from 2015 to 2024 exhibited evolutionary characteristics of overall optimization, reduced regional disparity, and strengthened key nodes. The development of tourist attractions and transportation infrastructure served as the primary drivers of improved accessibility. Guided by policy initiatives and driven by tourism market demand, the number of high-grade tourist attractions in the province increased by 110 between 2015 and 2024, including 104 newly designated 4A-level and 6 additional 5A-level attractions. This growth in the number of high-grade tourist attractions significantly enhanced regional accessibility [37]. During the 13th Five-Year Plan period, the total highway mileage in Anhui Province reached 236,000 kilometers, ranking seventh nationally. The province’s trunk road network, comprising railway, expressways, national highways, and provincial roads, was further optimized to form an efficient linkage system that connects scenic areas, transportation hubs, and tourist source regions [38]. However, in some regions, bottlenecks remain in the extension of terminal transportation networks. Addressing these challenges is essential for strengthening the tourism transport support system and advancing high-quality regional tourism development.

#### 3.1.2. Tourist attractions accessibility of the county-level administrative units.

**Fig 5:**
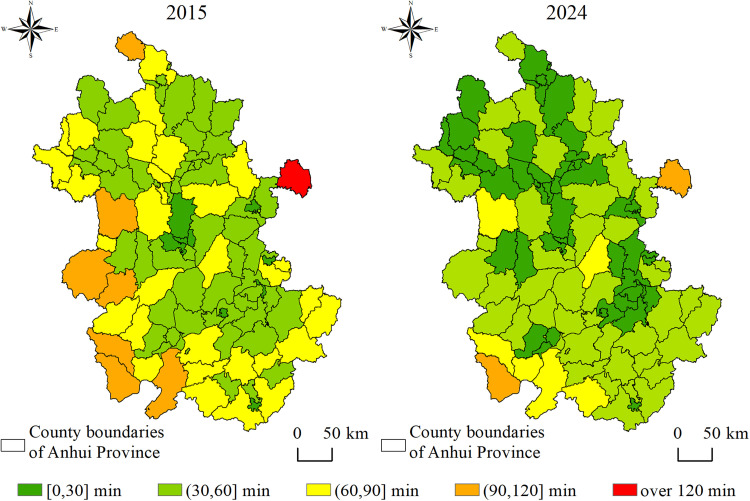
Tourist attractions accessibility of county-level administrative units.

Through zonal statistics, the accessibility of high-grade tourist attractions in each county-level administrative unit of Anhui Province is assessed (Fig 5). From 2015 to 2024, the accessibility levels across areas exhibited clear characteristics of overall improvement and gradient optimization. In 2015, 62.5% of counties had accessed to high-grade tourist attractions within 60 minutes, with these areas primarily clustered around the cores of prefecture-level cities and a few neighboring counties, indicating a spatial pattern characterized by central agglomeration and limited peripheral diffusion. In contrast, counties such as Huoqiu, Jinzhai, Taihu, Susong, Dongzhi, and Tianchang recorded accessibility time exceeding 90 minutes, exhibiting poor tourist attractions accessibility. By 2024, the proportion of counties with the time of accessibility within 60 minutes had increased to 92.3%. High-accessibility zones expanded beyond their original cores, demonstrating a pattern of contiguous spatial extension and broader regional coverage. Only Tianchang and Susong still had accessibility time exceeding 90 minutes.

In northern Anhui, where plains dominate and natural landscape resources are relatively scarce, recent years have seen the emergence of high-grade tourist attractions through the development of cultural tourism resources such as Chu–Han heritage, Huaihe River culture, and the philosophical legacy of Laozi and Zhuangzi. Coupled with improvements in intercity transportation infrastructure, tourist attractions accessibility in this region has been significantly enhanced. This is consistent with recent studies suggesting that the development of cultural heritage resources in less scenic areas, supported by transport improvements, can enhance tourism accessibility [39].

In the central region of Anhui Province, with Hefei serving as the core, zones of high accessibility have progressively expanded to encompass surrounding prefecture-level cities such as Lu’an, Chuzhou, Ma’anshan, and Wuhu. As part of the Hefei Metropolitan Circle, this area benefits from a high level of economic development, a well-integrated transportation network, and robust tourism demand, all of which have jointly contributed to enhanced accessibility to high-grade tourist attractions. High accessibility spreads from Hefei toward neighboring cities, indicating a spatial spillover effect. Such a trend is consistent with evidence that metropolitan transportation hubs enhance tourism accessibility by driving economic integration and facilitating infrastructural connectivity [40].

Southern Anhui’s unique topography, geomorphology, and cultural-historical backdrop endow the region with rich and diverse tourism resources, positioning it as a core area of the province’s tourism industry. In earlier development stages, complex terrain limited transportation infrastructure, leading to a high proportion of low-accessibility areas for tourist attractions. However, the construction of High-speed railway, expressways and scenic roads (such as the South Anhui Sichuan–Tibet Line) has significantly improved regional tourist attractions accessibility [41].

#### 3.1.3. Direction, extent and center of temporal distribution of accessibility.

**Fig 6:**
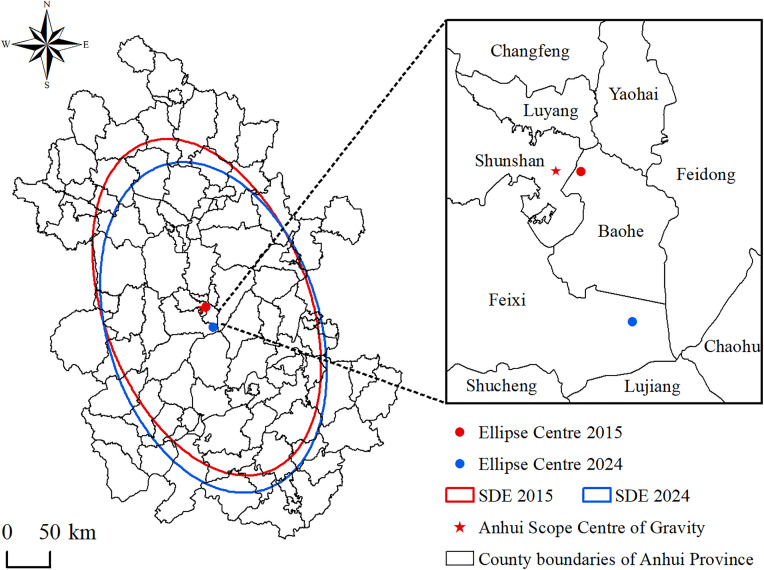
Standard deviational ellipse of tourist attractions accessibility.

Based on the tourist attractions accessibility of county administrative units, the direction, scope, and spatial center changes of the distribution of the accessibility are further explored by the standard deviation ellipse method (Table 3 and Fig 6). In terms of directionality, the azimuth angles of the ellipses derived from county-level accessibility were 156° and 160° in 2015 and 2024, respectively. The directional distribution of accessibility was largely consistent with the administrative outline of Anhui Province, presenting a northwest–southeast orientation. The ellipticity values were 1.7 in 2015 and 1.6 in 2024, indicating a weakening trend in directionality. Regarding spatial extent, the areas enclosed by the ellipses based on the time of accessibility were 76,553 km^2^ and 76,561 km^2^ in 2015 and 2024, respectively, accounting for approximately 55% of the province’s total area. The proportion of the accessibility time falling within these elliptical regions reached 68%, suggesting a pronounced spatial agglomeration of accessibility. As for distribution centers, the centroid coordinates of the ellipses in 2015 were 117°26′73″E and 31°82′50″N, closely aligned with the geographic center of Anhui Province. In 2024, the centroid shifted southeastward to 117°35′07″E and 31°61′39″N, indicating that improvements in accessibility were more significant in the western and northern regions of the province compared to the east and south. In southern Anhui, mountainous terrain results in higher development costs and greater challenges in constructing both tourist attractions and transportation infrastructure, which has slowed improvements in accessibility [42]. In contrast, northern Anhui, characterized by flat plains, has benefited from relatively low development costs. In recent years, improvements in transportation infrastructure and the growth of high-grade tourist attractions have significantly enhanced regional accessibility.

**Table 3:** Parameters related to standard deviational ellipse of tourist attractions accessibility.

Year	Length (km)	Area (km^2^)	Center X	Center Y	Major axis (km)	Minor axis (km)	Rotation (°)
2015	1033	76553	117°26′73′′E	31°82′50′′N	204	120	156
2024	1021	76561	117°35′07′′E	31°61′39′′N	197	123	160

### 3.2. Analysis of the coordinated development of supply and demand

#### 3.2.1. Description of demand indicator selection.

The scientific selection of tourism demand indicators in investigating the coupling relationship between the accessibility of high-grade tourist attractions and tourism demand requires consideration of both their theoretical relevance and data availability. Tourism demand encompasses both internal and external dimensions [43], each driven by distinct factors, and the selection of indicators should reflect these differences. From the perspective of internal tourism demand, under China’s current vacation system, short holidays of 2–3 days are common, leading many residents to engage in short-distance, local travel [44]. The population size of a region determines the base of potential tourists [43], while the level of economic development influences residents’ motivation and ability to travel [45]. Together, these constitute the core determinants of internal tourism demand. Therefore, the number of permanent residents and per capita GDP are selected as key indicators for quantifying internal tourism demand at the regional level. The former reflects the scale of the potential tourist market, while the latter reflects residents’ consumption capacity for tourism. In terms of external tourism demand, travel agencies, which serve as key hubs in the tourism industry chain, directly reflect the attractiveness of regional tourism resources and indirectly indicate the scale of inbound demand [46]. Similarly, hotels, as core infrastructure supporting tourism accommodation, reflect the capacity to host tourists from outside the region [47]. A higher number of hotels generally suggests greater tourism demand volume and intensity. Therefore, the number of travel agencies and hotels are selected as representative indicators of external tourism demand.

**Fig 7:**
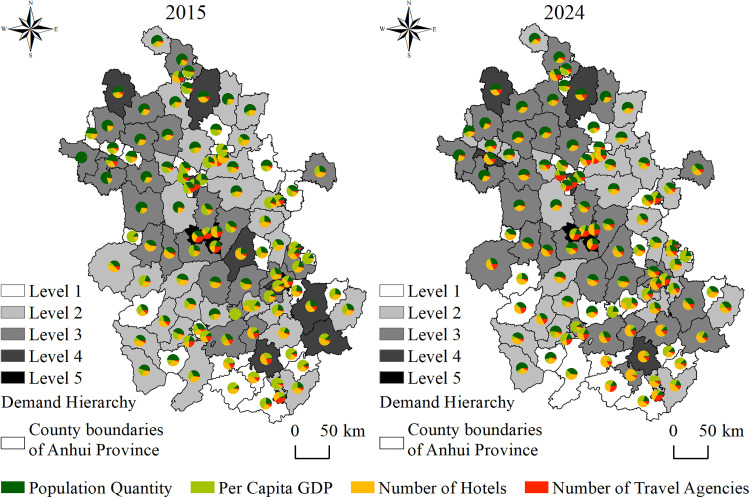
Regional tourism demand indicators and comprehensive evaluation.

The specific conditions of each indicator and the classification of tourism demand levels are illustrated (Fig 7). From 2015 to 2024, the tourism demand hierarchy across various regions in Anhui Province remained relatively stable. Among them, Luyang, Baohe, Shushan, and Yaohai exhibited the highest tourism demand levels, while Yeji, Datong, Jingde, and Bagongshan remained at the lower end of the spectrum. The remaining regions mostly fell within Level 2 to Level 4. From the perspective of specific indicators, the northern region of Anhui Province has a relatively larger population, while the southern region has a comparatively higher number of hotels.

#### 3.2.2. Spatial differentiation of coupling coordination.

**Fig 8:**
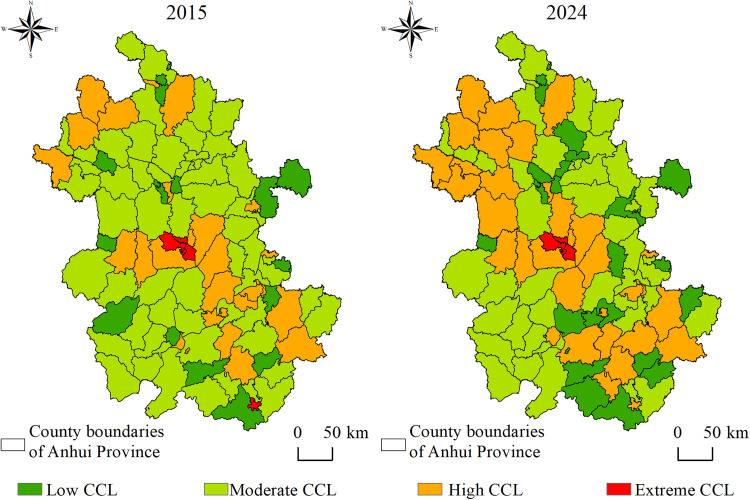
Spatial pattern of supply-demand coupling coordination level.

The coupling coordination degree between the accessibility supply of high-grade tourist attractions and the level of tourism demand in each county and district of Anhui Province was calculated using a coupling coordination model (Table 4 and Fig 8). The results were classified into four categories: low coupling coordination (Low CCL), moderate coupling coordination (Moderate CCL), high coupling coordination (High CCL), and extreme coupling coordination (Extreme CCL), based on standard thresholds widely applied in coupling coordination degree research [48]. In 2015 and 2024, the regional average coupling coordination degrees were 0.43 and 0.42, respectively. The overall spatial pattern was characterized by the predominance of Moderate CCL areas and a dynamically adjusting coordination structure.

**Table 4:** Supply and demand coupling coordination level distribution statistics.

CCL	D-value	2015		2024	
		Number of counties	Proportion (%)	Number of counties	Proportions (%)
Low CCL	0 < D ≤ 0.3	16	15.4	23	22.1
Moderate CCL	0.3 < D ≤ 0.5	58	55.8	45	43.3
High CCL	0.5 < D ≤ 0.8	26	25.0	33	31.7
Extreme CCL	0.8 < D ≤ 1	4	3.8	3	2.9

The spatial extent of Low CCL areas expanded, with increasingly pronounced marginal characteristics. In 2015, there were 16 Low CCL areas, accounting for 15.4% of the total. By 2024, this number had increased to 23 (22.1%), reflecting both the persistence of previously low-coordination regions and the downgrading of some formerly moderately coordinated areas. The extent of Moderate CCL areas contracted, accompanied by significant internal differentiation. From 2015 to 2024, the number of Moderate CCL regions declined from 58 to 45, indicating a redistribution toward both lower and higher coordination levels. The number of High CCL areas increased, with clustering characteristics becoming more evident. Over the study period, the number of High CCL regions rose from 26 to 33, primarily concentrated in areas with both abundant tourism resources and strong market vitality. The scale of Extreme CCL areas declined, although core areas remained relatively stable. In 2015, four regions were categorized as Extreme CCL: Shushan, Baohe, Luyang, and Tunxi. In 2024, Shushan, Baohe, and Luyang maintained extremely high coordination levels, benefiting from Hefei’s role as a hub of tourism services and its strong consumption agglomeration effect. In contrast, Tunxi dropped out of the Extreme CCL category, primarily due to intensified competition in regional tourism development, which led to the diversion of its resource advantages by surrounding areas.

#### 3.2.3. Change in the type of coupling coordination.

To analyze the spatial evolution characteristics of the supply–demand coupling coordination of high-grade tourist attractions, both a traditional Markov transition probability matrix and a spatially conditioned Markov transition probability matrix were constructed. The four types of coupling coordination (Low CCL, Moderate CCL, High CCL, and Extreme CCL) are represented by *k* = 1, 2, 3, and 4, respectively. Transitions from lower to higher levels are defined as upward transfers, while those from higher to lower levels are considered downward transfers. The traditional Markov transition probability matrix of coupling coordination levels between the accessibility of high-grade tourist attractions and tourism demand in Anhui Province from 2015 to 2024 (Table 5). According to the calculation results, the diagonal values of the matrix are all greater than the off-diagonal values, indicating that the transitions in coupling coordination types exhibit strong temporal stability. The probability of remaining in the same state is relatively high, with the High CCL type showing the highest stability, maintaining its original classification in the next period with a probability of 76.9%. The highest transition probability between different coordination levels is 25%, occurring between adjacent categories, specifically from Low CCL to Moderate CCL and from Extreme CCL to High CCL. In contrast, the probability of leap-level transitions is relatively low, with the highest being only 3.8%, representing a downward cross-level shift from High CCL to Low CCL. No upward cross-level transitions were observed during the study period.

**Table 5:** Traditional Markov transfer matrix (*k* = 4).

*t*_*i*_/*t*_*i*_ + 1	*n*	Low CCL	Moderate CCL	High CCL	Extreme CCL
Low CCL	16	0.750	0.250	0.000	0.000
Moderate CCL	58	0.190	0.603	0.207	0.000
High CCL	26	0.038	0.192	0.769	0.000
Extreme CCL	4	0.000	0.000	0.250	0.750

By incorporating spatial lag conditions into the traditional Markov chain transition probability matrix, a spatial Markov transition probability matrix was constructed to examine the transition probabilities of coupling coordination levels between the accessibility of high-grade tourist attractions and tourism demand in Anhui Province under the influence of neighboring regions (Table 6). Compared with the traditional Markov model, the transition probabilities of coupling coordination levels exhibit substantial variation under different spatial lag contexts. When the spatial lag type is “1”, the probability of maintaining a low coupling coordination state reaches 100%, compared to 75% in the traditional model. This suggests that proximity to low-coordination regions increases the likelihood of maintaining a low-level coordination state. When the spatial lag type is “2”, the probability of transitioning from an extremely high to a high coupling coordination state reaches 100%, significantly higher than the 25% observed in the traditional model. This indicates that adjacency to moderately coordinated regions substantially increases the likelihood of downward transitions. When the spatial lag type is “3”, the probability of transitioning from low to moderate coupling coordination rises to 100%, up from 25%. Simultaneously, the probabilities of maintaining both high and extreme coordination states also reach 100%. These results imply that adjacency to highly coordinated regions not only increases the likelihood of sustaining high and extreme coupling levels, but also enhances the probability of upward transitions. Overall, these findings demonstrate that geographical background (GB) plays a significant role in influencing the state transitions of coupling coordination between accessibility and tourism demand in Anhui Province.

**Table 6:** Spatial Markov transfer matrix (*k* = 4).

GB	n	*t*_*i*_/*t*_*i*_ + 1	1	2	3	4	GB	n	*t*_*i*_/*t*_*i*_ + 1	1	2	3	4
1	1	1	1.000	0.000	0.000	0.000	3	1	1	0.000	1.000	0.000	0.000
	0	2	0.000	0.000	0.000	0.000		5	2	0.200	0.600	0.200	0.000
	0	3	0.000	0.000	0.000	0.000		6	3	0.000	0.000	1.000	0.000
	0	4	0.000	0.000	0.000	0.000		3	4	0.000	0.000	0.000	1.000
2	14	1	0.714	0.214	0.071	0.000	4	0	1	0.000	0.000	0.000	0.000
	53	2	0.189	0.623	0.189	0.000		0	2	0.000	0.000	0.000	0.000
	20	3	0.050	0.250	0.700	0.000		0	3	0.000	0.000	0.000	0.000
	1	4	0.000	0.000	1.000	0.000		0	4	0.000	0.000	0.000	0.000

## 4. Conclusion and discussion

### 4.1. Conclusion

This study explores the coupling coordination between the accessibility of high-grade tourist attractions and tourism demand in Anhui Province from 2015 to 2024, offering both theoretical insights and practical implications for tourism geography and regional planning.

(1) Between 2015 and 2024, Anhui Province experienced a substantial improvement in overall accessibility, with the average travel time to high-grade tourist attractions decreasing from 65 to 41 minutes and the extent of highly accessible areas expanding significantly. Spatial disparities in accessibility have narrowed, although marginal areas with persistent low accessibility still exist, particularly in southern mountainous regions.
(2) At the county level, the proportion of areas with accessibility time to high-grade tourist attractions within 60 minutes increased from 62.5% to 92.3%. The improvement in accessibility to high-grade tourist attractions in counties located in western and northern Anhui Province was greater than that in the eastern and southern regions.
(3) The coupling coordination relationship between tourist attractions accessibility and tourism demand exhibit a stable structure dominated by moderate and high coordination types. Markov chain analysis shows that most regions tend to maintain their original coupling status, and leap-level transitions are rare. Spatial Markov analysis further reveals that neighboring areas significantly influence transition probabilities. When adjacent to low or moderate coupling coordination areas, the probabilities of maintaining a low coupling state and experiencing downward transitions increase. In contrast, adjacency to highly coordinated areas increases the likelihood of maintaining high or extreme coordination states, as well as the probability of upward transitions.

These findings highlight the critical influence of spatial context on tourism development and offer empirical evidence for optimizing the spatial allocation of scenic areas and related infrastructure, which in turn promotes the high-quality development of regional tourism.

### 4.2. Discussion

Theoretically, this study enriches the application of spatial coupling theory in tourism contexts and contributes to the refinement of the theoretical system in tourism geography and spatial economics. Practically, the findings offer guidance for regional tourism transportation planning and the spatial layout of scenic attractions, while supporting the precise regulation and management of the tourism market.

Although both grid-based and county-level analyses reveal improvements in accessibility between 2015 and 2024, notable scale-dependent differences are evident. The grid-based approach captures fine-grained variations in travel time, particularly in peripheral and mountainous areas where fragmented accessibility persists. By contrast, county-level aggregation tends to smooth out such heterogeneity, which may obscure local bottlenecks and lead to an overestimation of coordination levels in transitional zones. These discrepancies highlight the importance of adopting multiscale approaches in accessibility and coupling studies to mitigate the risk of ecological fallacy.

The spatial Markov chain analysis demonstrates that geographical background (GB) significantly shapes the coupling coordination between the accessibility of high-grade tourist attractions and tourism demand in Anhui Province. These spatial spillover effects align with Tobler’s first law of geography and corroborate the findings of Solarin et al., which highlighted the role of spatial proximity in tourism performance diffusion [20]. The absence of leapfrog transitions further suggests that structural constraints, such as infrastructure and demand inertia, limit the pace of systemic reorganization [23]. Simultaneously, the existence of threshold effects is evident. Advancing from a lower to a significantly higher coordination level requires simultaneous enhancements in both accessibility and tourism demand, which are often difficult to achieve within a short time frame.

Interestingly, while central Anhui, particularly the Hefei metropolitan region, maintained extremely high coordination due to its dual advantage in tourism service hubs and demand agglomeration, southern and eastern regions lagged despite rich tourism resources. This contradiction may be attributed to terrain-induced transport difficulties and imbalanced market access. Previous studies have similarly noted that natural endowment alone does not guarantee high coupling performance without adequate support infrastructure and strategic demand stimulation [35].

Moreover, the observed expansion of low-coordination areas at the provincial margins, particularly in 2024, highlights the widening gap between core and periphery. This spatial divergence aligns with the theory of cumulative causation, wherein resource-rich or infrastructurally advanced regions continue to attract more investment and visitors, further enhancing their accessibility-demand synergy [49]. Conversely, underdeveloped counties face compounded disadvantages such as limited infrastructure, sparse tourism services, and weak demand, resulting in persistent low coordination states [50].

Despite offering meaningful insights into the coupling coordination between accessibility and tourism demand in Anhui Province, this study is subject to several limitations. Firstly, the accessibility assessment is primarily based on cost distance analysis using topographical conditions and transportation network, while effective in capturing general spatial patterns, may oversimplify real-world travel behavior, such as route preferences, traffic congestion, or multimodal transport integration. Future research may incorporate dynamic traffic data and time-dependent models to enhance accuracy. Secondly, the selection of tourism demand indicators, although grounded in theoretical justification and data availability, may not fully capture the complexity of actual visitor behavior, particularly with respect to seasonal variation, digital mobility patterns, or short-term events such as festivals or promotions. Incorporating real-time tourism flow data, such as mobile signaling or online platform usage, could yield more behaviorally sensitive coupling results.
